# Author Correction: A functional selection reveals previously undetected anti-phage defence systems in the *E. coli* pangenome

**DOI:** 10.1038/s41564-024-01724-8

**Published:** 2024-05-20

**Authors:** Christopher N. Vassallo, Christopher R. Doering, Megan L. Littlehale, Gabriella I. C. Teodoro, Michael T. Laub

**Affiliations:** 1 Department of Biology, Massachusetts Institute of Technology, Cambridge, MA USA; 2 Howard Hughes Medical Institute, Massachusetts Institute of Technology, Cambridge, MA USA

**Keywords:** Bacteriophages, Bacteria

Correction to: *Nature Microbiology* 10.1038/s41564-022-01219-4, published online 19 September 2022

In follow-up studies on the CmdTAC system identified in our paper, we discovered that the wrong toxin clone was used for the spotting assays in Fig. 5f. A corrected version is shown below as Fig. 1. We now find that producing CmdT alone is not highly toxic to cells. However, producing CmdT and CmdA together severely diminishes growth, while co-producing CmdC along with CmdTA largely restores plating viability. These results, along with additional studies of the CmdTAC system^1^, indicate that CmdA is likely required for the folding or stability of CmdT and that, together, CmdA and CmdC neutralize CmdT activity. The corrected data do not affect our primary conclusion that the CmdTAC system provides *E. coli* potent defense against T4 infection, nor does it impact any other conclusions in the paper. We apologize for any confusion our error created.

To reflect the new findings, the following changes were made to the text. In the fourth paragraph of the “Previously uncharacterized toxin-antitoxin-like systems” section, the text now reading “Inducing the expression of just CmdT, a previously uncharacterized toxin that has an ADP-ribosyltransferase-like domain, was not toxic, but co-expressing CmdT with the presumed antitoxin, CmdA, was toxic to cells. Co-expression of CmdT with both the putative antitoxin and chaperone components largely restored viability (Fig. 5f)” previously read “Inducing the expression of CmdT, a previously uncharacterized toxin that has an ADP-ribosyltransferase-like domain, was toxic with the presumed antitoxin, CmdA, only marginally improved viability. However, co-expression with both the putative antitoxin and chaperone components completely restored viability (Fig. 5f).” In the final paragraph of the same section, the sentence now reading “Toxicity of MqsR…” previously began “Similar to CMdTAC, toxicity of MqsR…”.

**Fig. 1:**
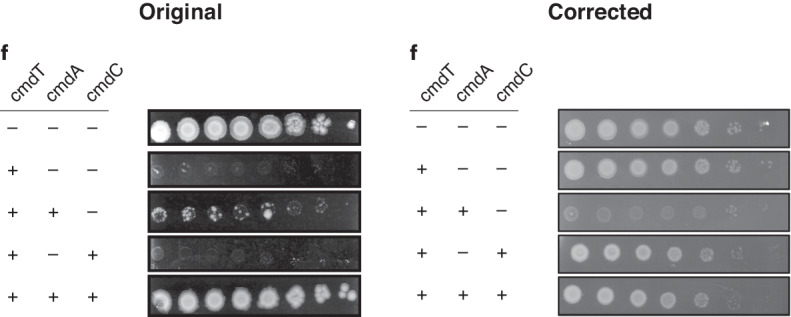
**Original and revised Fig. 5f**.
