# Supplementary material for: A functional selection reveals previously undetected anti-phage defence systems in the E. coli pangenome
Source: Nat Microbiol. 2022 Sep 19;7(10):1568–79. doi: 10.1038/s41564-022-01219-4 (PMC9519451; doi:10.1038/s41564-022-01219-4)
Supplement: Supplementary file 1 — Reporting Summary [file 41564_2022_1219_MOESM1_ESM.pdf]

nature portfolio

Corresponding author(s): Michael Laub

Last updated by author(s): July 25, 2022

Reporting Summary

Nature Portfolio wishes to improve the reproducibility of the work that we publish. This form provides structure for consistency and transparency in reporting. For further information on Nature Portfolio policies, see our Editorial Policies and the Editorial Policy Checklist.

Statistics

For all statistical analyses, confirm that the following items are present in the figure legend, table legend, main text, or Methods section.

n/a	Confirmed	
☐	☒	The exact sample size (*n*) for each experimental group/condition, given as a discrete number and unit of measurement
☐	☒	A statement on whether measurements were taken from distinct samples or whether the same sample was measured repeatedly
☐	☒	The statistical test(s) used AND whether they are one- or two-sided<br>*Only common tests should be described solely by name; describe more complex techniques in the Methods section.*
☒	☐	A description of all covariates tested
☒	☐	A description of any assumptions or corrections, such as tests of normality and adjustment for multiple comparisons
☐	☒	A full description of the statistical parameters including central tendency (e.g. means) or other basic estimates (e.g. regression coefficient) AND variation (e.g. standard deviation) or associated estimates of uncertainty (e.g. confidence intervals)
☐	☒	For null hypothesis testing, the test statistic (e.g. *F*, *t*, *r*) with confidence intervals, effect sizes, degrees of freedom and *P* value noted<br>*Give P values as exact values whenever suitable.*
☒	☐	For Bayesian analysis, information on the choice of priors and Markov chain Monte Carlo settings
☒	☐	For hierarchical and complex designs, identification of the appropriate level for tests and full reporting of outcomes
☐	☒	Estimates of effect sizes (e.g. Cohen's *d*, Pearson's *r*), indicating how they were calculated

Our web collection on statistics for biologists contains articles on many of the points above.

Software and code

Policy information about availability of computer code

Data collection	BLAST v. 2.11.0+ for homolog searches. HMMER3 v. 3.1b2 for domain searches. Taxonkit v. 0.8.0 to gather taxonomy information. MMseqs2 v. 13-45111 for protein sequence clustering. Custom code was generated, using these tools, to detect homologous proteins and their genome context from NCBI genomes. This code is publicly available at https://github.com/chrisdoering8197/phagedefense
Data analysis	HHsuite v. 3.1.0 for remote homology detection (includes HHblits and HHsearch). Prokka v. 1.14.6 for genome annotation. Roary v. 3.12.0 for pangenome analysis. FastTree v. 2.1.10 for phylogenetic tree construction. DefenseFinder v1.0 for prediction of known defense systems.

For manuscripts utilizing custom algorithms or software that are central to the research but not yet described in published literature, software must be made available to editors and reviewers. We strongly encourage code deposition in a community repository (e.g. GitHub). See the Nature Portfolio guidelines for submitting code & software for further information.

Data

Policy information about availability of data

All manuscripts must include a data availability statement. This statement should provide the following information, where applicable:

- Accession codes, unique identifiers, or web links for publicly available datasets
- A description of any restrictions on data availability
- For clinical datasets or third party data, please ensure that the statement adheres to our policy

All data generated or analyzed during this study are included in the published article (and its supplementary information files).

Field-specific reporting

Please select the one below that is the best fit for your research. If you are not sure, read the appropriate sections before making your selection.

☒ Life sciences ☐ Behavioural & social sciences ☐ Ecological, evolutionary & environmental sciences

For a reference copy of the document with all sections, see nature.com/documents/nr-reporting-summary-flat.pdf

Life sciences study design

All studies must disclose on these points even when the disclosure is negative.

Sample size	No sample size calculation was performed. Given large effect sizes, we chose to replicate experiments in triplicate as is standard to indicate reproducibility.
Data exclusions	Only representative sub-library peaks were included in Fig. 2C because the final identification of genes and their functions is confirmed in the subsequent figures.
Replication	All experimental findings were repeated at least twice. All reported results were successfully reproduced.
Randomization	No experimental groups or control groups were subjectively chosen and there are no covariates to control for as experiments were done in isogenic strains. No experiments required randomization.
Blinding	Blinding was not relevant because all data were obtained objectively and had strong effect sizes and raw data is reported in the manuscript. All comparative analyses were performed in isogenic strain backgrounds.

Reporting for specific materials, systems and methods

We require information from authors about some types of materials, experimental systems and methods used in many studies. Here, indicate whether each material, system or method listed is relevant to your study. If you are not sure if a list item applies to your research, read the appropriate section before selecting a response.

Materials & experimental systems

n/a	Involved in the study
☒	☐ Antibodies
☒	☐ Eukaryotic cell lines
☒	☐ Palaeontology and archaeology
☒	☐ Animals and other organisms
☒	☐ Human research participants
☒	☐ Clinical data
☒	☐ Dual use research of concern

Methods

n/a	Involved in the study
☒	☐ ChIP-seq
☒	☐ Flow cytometry
☒	☐ MRI-based neuroimaging
